# Application of augmented reality and surgical robotic navigation in total hip and knee replacement

**DOI:** 10.3389/fsurg.2025.1591756

**Published:** 2025-07-28

**Authors:** Xiuli Zhang, Yong Liu, Wen Luo, Yansu Guo

**Affiliations:** ^1^ The 1st Ward of Joint Surgery, Joint Surgery Department, Tianjin Hospital, Tianjin, China; ^2^ Orthropedic Department, Tianjin Hexi Hospital, Tianjin, China; ^3^ College of Artificial Intelligence and Data Science, Hebei University of Technology, Tianjin, China; ^4^ Beijing Geriatric Healthcare and Disease Prevention Center, Xuanwu Hospital, Capital Medical University, Beijing, China

**Keywords:** total hip replacement, total knee replacement, augmented reality, surgical robot, operative navigation

## Abstract

With advancements in computer vision, artificial intelligence, and other cutting-edge science and technologies, the focus of modern surgical technology has increasingly shifted towards intelligent, digital, minimally invasive, and precision approaches. Augmented reality (AR) technology and surgical robotics have emerged as significant research areas in total hip and knee replacement. Navigation systems, which are pivotal in both AR and robotic surgery, play a crucial role in guiding surgical operations using shared techniques. Recent developments in navigation systems for hip and knee replacement have focused on more natural, intelligent, and efficient methodologies. The use of AR and surgical robots for navigation has significantly enhanced the safety and accuracy of these procedures. Importantly, these technologies eliminate the need to implant positioning screws or other reference objects into the bone structure, thereby markedly reducing the risk of severe complications, such as lower limb pain and fractures. This study reviews the current applications, main challenges, and solutions associated with AR and surgical robot navigation systems for total hip and knee replacements.

## 1 Introduction

With the rapid advancement of artificial intelligence, precision instruments, computer vision, and other sophisticated technologies, robot-assisted surgery offers significant benefits, including minimal trauma, reduced risk of postoperative infections, and faster recovery. Currently, this technology is extensively applied in total hip and knee replacements (1–3). The primary advantage of surgical robots is their ability to combine the precision and dexterity of machines with the expertise of surgeons (4). The surgical robotic system incorporates multidisciplinary technologies, providing surgeons with capabilities for surgical planning, positioning, measurement, and enhanced visualization. These functions significantly improve the accuracy, safety, and reproducibility of surgical operations (5–8). Moreover, as medical imaging and computer technologies continue to advance, the concept of robot-assisted surgery has gained acceptance and recognition in the medical community. This concept encompasses various critical components, such as virtual surgical systems and surgical navigation systems (9). The fluency and precision of the surgical robot navigation system are crucial; for instance, the system involves the preoperative measurement of 3D data and intraoperative real-time registration of anatomical structures. Using an optical positioning device at the robot's end, it visualizes the patient's operative field, surgical instruments, and surgeon's posture in real-time using the visualization software, enabling precise navigation (10).

Augmented reality (AR) supplements real-world environments with computer-generated elements such as sound, video, and images, creating an enhanced sense of reality (11). This technology significantly improves the perception of the surgical environment, particularly for minimally invasive procedures. It not only helps doctors accurately position targets and improve patients' postoperative rehabilitation outcomes and quality of life but also provides a rich visualization of anatomical structures. This expansion of surgical visibility simplifies complex operations (12). In surgical applications, AR offers several main advantages: ① It seamlessly integrates real and virtual scenes, displaying them in real-time within the surgeon's field of view, which enhances the intuitiveness and locality of visual information. The system serves as an intraoperative navigational aid; ② A three-dimensional virtual model, reconstructed from two-dimensional tomographic imaging, compensates for limitations such as restricted visual space, poor image quality, and lack of depth perception; ③ When preoperative planning is conducted using a 3D virtual model, more precise and detailed surgical routes and areas can be designed. This approach also vividly portrays the spatial relationships among human tissues, surgical paths, and areas, reducing dependence on the surgeon's experience and imagination (13). During surgery, the AR surgical navigation (ARSN) system visually displays a translucent three-dimensional model of the target organ within the surgical field, aiding the surgeon in understanding the anatomy of the area (14). The application of AR technology in the surgical navigation of total hip and knee replacements is not constrained by factors such as cost, space, or equipment maintenance, thereby making clinical deployment more feasible.

In robotic surgical systems, the integration of AR technology enhances the system by compensating for reduced sensory perception, simplifying the surgical process, and enhancing both the safety and accuracy of operations (11). Currently, the surgical robot is equipped with a comprehensive human-computer interaction system that adequately supports the software and hardware requirements of the augmented reality navigation system through its own hardware and visualization capabilities. The incorporation of an augmented reality navigation system in robotic surgery not only facilitates easier operation but also offers greater cost-effectiveness (15). This paper reviews the current use of AR and surgical robot navigation systems for total hip and knee replacement, addressing the major challenges and solutions related to the deployment of ARSN systems in these procedures.

## 2 Augmented surgical navigation system

### 2.1 Navigation system based on touch control equipment

The touch control device operates in the manual input mode, requiring direct contact during use. Navigation systems that utilize touch control are exemplified by hand touch screens, which can function both as input devices to capture surgeons' movements and output devices to provide tactile feedback (16). The leading touch interaction technologies include 3D tactile feedback touchscreens and touch-sense tactile feedback technology (17–19). However, touch-based navigation systems present significant challenges in clinical surgery. Surgeons must often move away from the operation site to interact with the touchscreen, which disrupts the surgical workflow. This movement can lead to issues such as hand-eye coordination disruption and can compromise the surgical procedure. Consequently, AR navigation systems that rely on touch devices are seldom used in surgical settings.

### 2.2 Navigation system based on voice interaction

Speech recognition technology has evolved into a robust human-computer interaction method within AR systems, making voice the preferred mode of interaction in scenarios where traditional modes (such as hardware devices or touch control) are impractical (20). In AR settings, direct voice or voice-assisted interactions are possible. The focus is on the voice recognition engine, with major engines in the market including Speech API, Via Voice, and iFlytek (21). Voice interaction navigation systems in surgery offer several advantages: ① Voice interaction represents a novel, more convenient, and natural form of interactive control; ② As a close-range, non-contact type of control, it allows physicians to perform all control actions from a distance, thereby reducing contamination risks; ③ Recent studies have shown that integrating preset voice interaction commands can seamlessly connect the visualization system of surgery with the motion control system of the surgical robot. This integration enhances the cohesion of the robotic surgical system, simplifies the management of multiple subsystems, and ensures synchronization between visual information and robot motion (22–24).

### 2.3 Navigation system based on somatosensory interaction

A navigation system based on motion-sensing interaction technology utilizes optical capture principles and sensors or computer vision equipment to facilitate intraoperative navigation via the surgeon's gestures, eye movements, and other physical activities (25). For example, Ortega et al. used a micro-optical head-mounted display to show intraoperative perspective images in spinal fracture internal fixation surgeries. This system allows surgeons to use gestures to enlarge, shrink, and rotate images, thereby aiding in understanding the characteristics of the surgical site. Clinical trials involving 50 cases demonstrated that the micro-optical system significantly reduced the time surgeons spent looking at the image displays (26). Real-time position information of the identified surgical instruments is transmitted to the HoloLens device, enabling synchronous display of medical images, such as surgical instruments and lesion models. This provides visual displays of lesion images for doctors during surgery and facilitates lesion positioning and surgical instrument navigation through gesture interactions (27).

Owing to their convenience and robust interaction, somatosensory-interaction-based surgical navigation systems are becoming the primary development direction for total hip and knee replacement augmented reality navigation systems. Fotouhi et al. (2018) proposed an AR solution for positioning the acetabular cup in total hip arthroplasty. This system plans the placement of an acetabular cup using two-dimensional x-ray images taken after acetabular reaming combined with three-dimensional AR visualization technology. It aligns point-cloud data with the planned path to accurately position the cup (28). However, the study noted that the initial positioning of the pelvis in total hip replacements often reduces the accuracy of AR system image registration (29). To address this issue, researchers have attempted preoperative pelvic radiography before AR system image registration during THA by adjusting the angle of the operating table to enhance the accuracy of the AR navigation system (30). Total hip replacement poses greater challenges in special cases such as ankylosing spondylitis, where traditional surgical methods typically require repeated fluoroscopy to determine the position of the hip prosthesis. Recent studies have shown that AR can facilitate 3D imaging, enabling surgeons to observe the hip joint anatomy in real time (31). Although studies are limited, existing research confirms that AR navigation technology can achieve more precise tilt and anteversion angles in total hip arthroplasty, potentially extending the lifespan of the prosthesis (32–34).

## 3 Robot navigation system for total hip and knee replacement surgery

Owing to the rigid nature of bones compared with that of other body parts, the application of surgical robots in joint surgery presents challenges, making the joint replacement robot system a focal point in orthopedics (35). For total knee replacement, the navigation system acts as a guide, offering preoperative planning and assisting doctors in precisely locating the implant position (36). The primary purpose of the navigation system is to facilitate operations and enhance surgical safety, making it a crucial component of robotic surgical systems.

The Mako knee joint replacement surgical robot system, developed by Stryker, pioneered the integration of an optical external tracker into the surgical tool navigation system, accurately facilitating surgical navigation during operations (37). In 2016, Bell successfully performed a total knee replacement surgery with the assistance of the Mako surgical robot. The experimental results indicated that compared with traditional methods, the installation error in robot-assisted total knee replacement was maintained within 2° in patients (38). The French company Medtech introduced the ROSA surgical robot, which consists of a mechanical arm and navigation system featuring a 6-DOF robotic arm with tactile sensors and a touch-screen system workstation integrated into a single platform. The navigation system utilizes touch interaction technology for robot registration, target trajectory planning, and intraoperative image guidance, all of which are completed by the platform (39). Several studies confirmed that the precision of joint prosthesis placement in robot-assisted total knee replacement exceeds that in traditional surgery. Rossi et al. reported that the average error for the femoral inversion angle and femoral inclination angle in robot-assisted procedures was 0.5 ± 0.6°, better than the AR navigation system group's 0.59 ± 0.55° and 0.7 ± 0.75° (40). Hampp et al. found that the mean error value for the femoral inversion angle in robot-assisted total knee arthroplasty (0.6 ± 0.3°) was significantly lower than in the conventional surgery group (1.1 ± 1.6°) (41).

In recent years, China has made significant advancements in the development of joint-replacement surgical robots. Several domestic models such as HURWA, ARTHROBOT, Bone, and Honghu have been introduced into the market, showing promising medium- and long-term clinical outcomes. Specifically, in the HURWA robotic surgery system, the accuracy of the lower limb force line reconstruction in total knee arthroplasty patients reached 81.2%, compared with only 63.5% in the traditional surgery group. This system can safely and reliably support the execution of robot-assisted total knee replacements. The robot integrates cutting-edge technologies such as virtual fixtures and shared control, enabling precise surgical operations (42).

## 4 Clinical outcomes from AR and robot navigation systems

### 4.1 Clinical outcomes from AR in total hip and knee replacement

The effect of AR in knee replacement surgery is influenced by the early negative effect of cutting errors. Some researchers have attempted a volume-subtraction technique to decrease cutting error (43). The results showed a significant improvement in alignment accuracy from 0.55–0.64 to 0.40–0.55 mm, while the processing time decreased from 12–13 fs/s to 9–10 fs/s (43). Valenti (44) developed an automatic method for recovering the pose of the knee in 3D and ensured the implantation of the prosthesis in the correct pose. The proposed system allows successful placement of the prosthesis within the planned coordinates, with minor errors of 2 cm and 2°. In THA, the differences in radiographic inclination were significantly smaller in the AR-based portable navigation system than in the accelerometer-based portable navigation system (2.5 ± 1.7 vs. 4.6 ± 3.1) (45). Even when THA is performed with the patient in the lateral decubitus position, the AR-HIP system allows the surgeon to create a 3-dimensional coordinate system for positional information for formation of the acetabular cup with the patient in the supine position, which is very convenient for surgeons (46). One study confirmed that the measurement angle using AR-HIP was significantly more accurate in terms of radiographic anteversion than using a goniometer 3 months after THA (2.7 vs. 6.8) (47). However, the study showed that the procedure time was longer in the navigation group (95 min vs. 57 min) (48). In terms of mid-term postoperative outcomes, no differences were observed between the AR-based portable hip navigation system and the conventional technique in improvement in HOOS (27 ± 17 vs. 28 ± 19) at 6 months after THA (48).

### 4.2 Clinical outcomes from robot navigation systems in total hip and knee replacement

A prospective, randomized controlled trial showed that the operation time in the robot-assisted TKA group was significantly longer than that in the traditional TKA group, and no significant difference was observed in intraoperative blood loss between the two groups (49). Three months after surgery, gait analysis showed that the flexion and extension angles in the RATKA group were significantly larger than those in the traditional TKA group and that the lateral tibial component was significantly smaller in the RATKA group than in the traditional TKA group, which was closer to the ideal value (49). Robot-assisted TKA is associated with reduced postoperative pain, decreased analgesia requirements, decreased reduction in postoperative hemoglobin levels, shorter time to straight leg raise, decreased number of physiotherapy sessions, and improved maximum knee flexion at discharge compared to those in conventional TKA (50). The median time to hospital discharge in robot-assisted TKA was 77 h compared with 105 h in conventional TKA (50). Two years after TKA, the robot-assisted group displayed a trend towards higher SF-36 QoL scores, with significant differences in SF-36 vitality and role-emotional (51). A systematic review and meta-analysis showed that RATKA resulted in a better Knee Society Score than mTKA in short-to mid-term follow-up (52). Compared with conventional TKA, the RATKA improved component positioning and alignment (−1.30 to −3.02 degrees) (53).

## 5 Augmented reality and major problems facing surgical robot navigation systems

### 5.1 Technical challenges

#### 5.1.1 Data transmission delay

The primary purpose of the navigation system is to provide the surgeon with a three-dimensional model that displays the anatomical situation and any changes in the patient's lesions, thereby facilitating precise pre-surgical planning. Additionally, the system presents real-time updates on the patient's surgical site and ongoing operation, allowing the surgeon to assess the surgical outcome as it unfolds. During surgery, it is crucial to ensure that the model is transmitted and accurately projected to the surgeon-specified location. The hardware must rapidly process and update the model information in response to the surgeon's movements and adjustments, thus demanding high real-time performance in model transmission and feedback. The speed of the processor and communication within a computer system can introduce delays in data transmission, which pose significant challenges to the clinical implementation of this technology. A single-center clinical randomized study demonstrated that using the AR navigation system in total knee arthroplasty (TKA) added an average of 5 ± 1 min to the procedure. However, the precision of the surgical incision and prosthesis placement was superior to that in the traditional surgery group. These findings underscore the potential benefits of AR navigation technology in arthroplasty (54).

#### 5.1.2 Information processing capability of the navigation system

Both AR navigation systems and surgical robot navigation systems rely on various computer vision technologies to register the 3-dimensions (3D) model of the patient's site with real scene images. During surgery, these systems facilitate surgeon interactions and guide operations, allowing the real-time evaluation of surgical outcomes. Consequently, the internal processor of a surgical robot requires robust information processing capabilities to ensure that relevant data can be accessed in real-time. This requirement poses significant challenges for the processor and memory of surgical robot systems. As surgical complexity increases, the number of system model parameters also increases, adding to the computational load on the internal processor. This escalation demands enhanced capabilities from graphics processing units (GPUs). Future research will need to explore various optimization techniques, such as Bayesian hyperparameter optimization, integration of the transformer self-attention mechanism, and gating units, to enhance the efficiency of the internal processor model and ensure the precision and effectiveness of intraoperative navigation.

#### 5.1.3 Image registration and tracking

The use of navigation and nail positioning is a prerequisite for operating navigation systems during joint replacement surgery. Surgical robot navigation systems typically rely on external tracking mechanisms that require fixed positioning benchmarks (navigation and positioning nails) attached to bone and surgical instruments to identify and guide robotic arm movements (55). In systems that use reflective balls for optical tracking, a benchmark must be implanted and secured to the patient's tissue, which can be invasive and physically disruptive (56). This additional step alters the standard surgical workflow, introduces additional procedural steps, extends the operation time, and increases surgical risks. Ossendorf and Jung described the world's first robot-assisted total procedure in 2006 (57). Bonutti et al. later confirmed that being overweight and obese are significant risk factors for positioning needle fractures after robot-assisted total knee arthroplasty (58). Jung et al. found that repeated intraoperative implantation of navigation and positioning nails could lead to abnormal lower limb pain (55).

The static pose needs to be further updated by dynamic target tracking because the bone inevitably moves during total hip or knee replacement. Meanwhile, in a practical setup, such a markerless tracking and registration algorithm in AR can produce unreliable registration results, particularly in rotation (59). During tracking, the initially registered bone pose is continuously updated by monitoring the optically tracked dynamic reference frame movement, which results in a long workflow (60) and possible human-induced errors (61). Markerless tracking and registration algorithms have been proposed for knee surgery (62). The segmented femur points were then registered to a pre-scanned model of the corresponding limb using the iterative closest point method in real time to obtain the spatial knee pose (63). The best-reported registration accuracies are 6.66° and 2.74 mm when the target is held static, which is not acceptable for clinical applications (64).

### 5.2 Clinical challenges

Even though the application accuracy can be improved by different methods to allow for optimized initial patient-to-image registration, accuracy is constantly decreasing throughout the surgical procedure and, in the worst case, might even lead to an unacceptable mismatch in AR or surgical robot navigation systems (65). Most commercially available navigation systems enable intraoperative landmark-based registration updates to overcome alterations in spatial relationships of the reference array (66). Therefore, readily available and uniquely identifiable landmarks, such as positioning nails, can be acquired and used at any time to restore accuracy if a discrepancy is observed in paired-point registration (66). However, loss of accuracy up to the point of acquisition (e.g., skin incision, interchange of reference arrays) cannot be compensated for in this way, and the same accounts for the effects of knee shift. Moreover, due to the lack of an intraoperative feedback system, especially fully automatic robots, any malfunction during the operation has serious consequences (67). Currently, most joint replacement surgery robots lack intraoperative sensory feedback systems, such as touch, toughness, and temperature, which can easily lead to accidental injuries (68).

### 5.3 Economic factors

The application of AR and robot assistance comes at an additional cost compared with that of the conventional technique. Although a short-term cost analysis on RA-TKA has been performed, a 90-day episode-of-care cost analysis showed that reduced costs were possible in robot-assisted TKA compared with that of the conventional technique because of fewer readmissions and economically beneficial discharge destinations (69). Markov decision analysis confirmed that a calculated surgical volume of at least 253 cases per robot per year is needed to prove cost-effectiveness, considering predetermined parameter values (70). At a minimum follow-up of 10 years, the study found no differences between robot-assisted TKA and conventional TKA in terms of functional outcome scores, aseptic loosening, overall survivorship, and complications; however, considering the additional time and expense associated with robot-assisted TKA, researchers cannot recommend its widespread use (71). Meanwhile, another study found that 51% of robotic UKA manuscripts were industry-funded or had authors with financial conflicts of interest compared with 29% of non-robotic UKA papers (72).

### 5.4 Surgeon training and cognitive load

A learning curve of operative time has been found with the introduction of RATKA surgery, which varies based on the surgeon's experience and volume (73). Many studies have reported the learning curves associated with the introduction of a different number of RATKA cases, ranging from 6–43 cases (36, 74, 75). One study found that the introduction of the RATKA system was associated with a learning curve for an operative time of 8.7 cases (76). The operative times were similar between the RATKA and conventional TKA groups. The short learning curve implies that the RATKA system can be adopted relatively quickly by a surgical team with minimal risk to patients (76). Meanwhile, a study showed that haptic feedback by AR can guide participants using a tool to submillimeter and subdegree accuracy with little training (77).

## 6 Augmented reality-robotic surgical navigation system solutions

Total hip and knee replacement surgery involves not only bone tissue but also the surrounding soft tissue. Surgical procedures lead to shifts in tissue position, which increases the complexity of image tracking. Researchers have explored various solutions to address image registration and tracking challenges in navigation systems. Mur-Artal et al. (78) proposed the ORB-SLAM 2 (Oriented FAST and Rotated BRIEF Simultaneous Localization and Mapping 2) method, which effectively eliminates accumulated errors in image tracking and can automatically relocalize after tracking failures. Bescos et al. (79) introduced a deep learning-based surgical scene segmentation method capable of identifying real-time soft tissue displacement in a dynamic environment, thereby improving synchronous localization and mapping accuracy.

The AR reality joint replacement surgical robot system effectively overcomes the reliance on navigation positioning nails for intraoperative image registration and tracking by utilizing 2D/3D medical image registration technology. The system accurately guides the surgical robot during the procedure by aligning preoperative 3D images with real-time intraoperative 2D images. Liao et al. (80) leveraged the characteristics of x-ray 2D images. They employed digitally reconstructed radiographic technology to directly measure registration errors between 3D CT images and intraoperative x-ray images, thereby enhancing positioning accuracy.

To address the issues of image transmission delays and prolonged system feedback processing times caused by the high computational complexity of the internal model in surgical robot navigation systems, researchers have explored lightweight optimization of deep learning algorithms. These improvements enhance the model's information processing capabilities, reduce system feedback time, and ensure real-time data and image transmission. Researchers successfully achieved 3D/2D image registration of spinal joints using a reinforcement learning algorithm, demonstrating a mean square error of just 0.0858 and an average processing time of only 6.54 s (81). Additionally, studies have confirmed that segmenting intraoperative 2D images using a DeepLabv3+ neural network, enhanced by an attention mechanism and contour extraction, significantly improves registration accuracy with preoperative 3D images (82).

## 7 Future and outlook

The AR–surgical robot navigation system significantly improves the safety and accuracy of total hip and knee replacement procedures. Unlike traditional methods, it eliminates the need for implanting positioning screws or other reference objects on the bone structure, effectively reducing the risk of serious complications such as lower limb pain and fractures. Current research on AR surgical robot navigation systems in joint surgery primarily remains at the stage of technical feasibility exploration (83). Considerable heterogeneity exists among studies. Designing multicenter, randomized controlled trials in the future should include primary clinical outcomes such as operation time, intraoperative blood loss, lower limb alignment, and prosthesis position, as well as secondary clinical outcomes such as visual analog scale scores, range of motion, function scores, and gait analysis. Meanwhile, as science and technology continue to advance, reference should be made to robot safety standards, such as ISO 13482, to standardize the clinical application of surgery robots and augmented reality (84, 85).
